# Evaluating effects of tissue type, preservation method, and decomposition on DNA quality to support genetic sampling in stranded small cetaceans

**DOI:** 10.1038/s41598-026-41686-x

**Published:** 2026-04-28

**Authors:** Miguel L. Grilo, Miguel Leal e Rigor, Andre E. Moura, Karen Avellaneda, Stephanie Gross, Joana I. Robalo, Filipe R. Ceia, Ursula Siebert

**Affiliations:** 1 Institute for Terrestrial and Aquatic Wildlife Research, University of Veterinary Medicine Hannover, Foundation, 25761 Büsum, Germany; 2 RALVT— Lisbon and Tagus Valley Stranding Network, ISPA—Instituto Universitário de Ciências Psicológicas, Sociais e da Vida, Lisbon, 1149-041 Portugal; 3 MARE—Marine and Environmental Sciences Centre/ARNET—Aquatic Research Network, ISPA—Instituto Universitário de Ciências Psicológicas, Sociais e da Vida, Lisbon, 1149-041 Portugal; 4 Department of Life Sciences, University of Coimbra, Coimbra, 3000-456 Portugal; 5 Faculty of Biology, University of Gdańsk, Gdańsk, 80-308 Poland; 6 Department of Life Sciences, University of Coimbra, MARE – Marine and Environmental Sciences Centre / ARNET - Aquatic Research Network, Coimbra, 3000-456 Portugal

**Keywords:** Cetacean, Genetics, DNA, Ethanol 96%, Freezing, Stranding, Biological techniques, Genetics, Molecular biology, Zoology

## Abstract

Effective management decisions rely on accurate insights into species’ population structure, with high-quality genetic material being essential. Stranded cetaceans are an important source of samples, but meeting quality standards remains challenging. Despite their frequent use in conservation genetics, no controlled studies have compared DNA yield and quality across tissue types and preservation methods. This is further compounded by cetaceans uniquely derived physiology, meaning standards obtained from other mammals might not apply. In this study, we compared DNA concentration, purity, and integrity when extracted from skin, muscle, and blubber samples preserved in 96% ethanol or frozen at − 20 °C. Samples were obtained from a tissue bank of stranded dolphins and classified into five decomposition condition categories following international guidelines. All samples underwent standardized protocols for DNA extraction, quantification, and quality control. Statistical analyses evaluated the influence of tissue type and preservation method on DNA quality. Skin consistently produced the highest DNA concentrations and acceptable purity levels, even in advanced decomposition stages. DNA integrity declined with decomposition, except in muscle, which retained higher integrity values in later stages. Both 96% ethanol and freezing at − 20 °C produced good results in early decomposition stages, while freezing was particularly important for maintaining DNA integrity in muscle in more advanced decomposition. Based on these findings, we propose two frameworks: one guiding researchers according to sequencing requirements, and another simplified version for stranding networks. These results contribute to standardized sampling protocols and improve the reliability and comparability of genetic analyses from stranded cetaceans.

**Supplementary Information:**

The online version contains supplementary material available at 10.1038/s41598-026-41686-x.

## Introduction

Stranding networks are invaluable sources of biological data and material for marine mammals. Such data helps gaining a comprehensive knowledge of their population dynamics, which is an essential requirement for several downstream applications including management decisions for effective marine mammals conservation. However, gathering such information is particularly challenging due to their highly mobile and often elusive nature^1^. In this regard, genetic analysis provides a powerful tool for studying cetacean ecology, evolution, and conservation. Genetic data facilitate the assessment of population structure, connectivity, and gene flow, which are crucial for identifying distinct populations and their migration patterns^2,3^. Moreover, genetic studies contribute to understanding the evolutionary relationships among species, aiding in reconstructing their phylogenetic history^4^. From a conservation perspective, genetic markers help delineate management units and identify demographic groups that may be more vulnerable to environmental changes or anthropogenic threats^5^. The use of genetic techniques also extends to health assessments, where molecular methods can evaluate the genetic basis of disease resistance or susceptibility in cetacean populations^6^. Such insights inform targeted conservation strategies, ensuring the long-term viability of these species in the face of anthropogenic pressures, habitat degradation, and climate change^1^.

Despite the value of genetic research in cetacean conservation, acquiring high-quality samples from live animals presents logistical, financial, and ethical challenges^7–9^. Sampling of internal tissues requires highly invasive techniques that are not ethically feasible for marine mammals. Minimally invasive techniques such as skin and blubber biopsies still require specialized equipment, trained personnel, and consideration of potential stress inflicted on the animals^8^. Dead stranded cetaceans, on the other hand, are not subject to such limitations and offer a unique research opportunity by providing access to a wide range of tissue types, including internal organs and blood, which are often unavailable in live sampling^9^. Accordingly, over the last decades, cetacean researchers have relied on stranding material to obtain DNA for a range of genetic studies^3,10,11^. However, a significant limitation is the degradation of genetic material due to decomposition, which can hinder the accuracy of analyses^12–14^. Environmental factors, such as temperature, salinity, contamination, and scavenger activity, further complicate sample preservation^15^. Therefore, researchers working on DNA from stranded samples commonly experience considerable variation in success rate between samples, involving unavoidable waste of time and lab resources processing samples whose DNA is not usable for many downstream analyses.

To mitigate these challenges, standardized protocols for tissue collection and preservation from stranded cetaceans must be refined to optimize resulting DNA integrity and help ensuring reliable results are obtained ahead of any experimental work. While stranding networks generally adhere to standardized guidelines established by national programs or international reference societies^9,16,17^, they do not all follow unique tissue sampling protocols for downstream DNA analyses. Such standardization is necessary to ensure sample quality consistency, as it enables uniform data collection which is vital for comparing and integrating data from different regions and networks^18,19^. Sampling frameworks should cover both methodological approaches to tissue collection and preservation strategies based on the animal’s decomposition. Furthermore, sampling and storage guidelines should offer options tailored to tissue banks facing economic and logistical (e.g. field conditions) challenges.

While dedicated sampling guidelines for genetic analysis have been developed^19,20^, including a thorough sampling framework covering sample type, storage, and genetic applications, they are not informed by a systematic comparative analysis of the efficiency of different preservation methods and tissue matrices regarding DNA quality parameters. Such comparisons have been conducted for other taxonomic groups^21^, but cetacean physiology is uniquely derived compared to terrestrial mammals, and therefore a dedicated study is required but is currently lacking. In this study, we assessed the influence of different tissue matrices (skin, blubber, and muscle) and preservation methods (96% ethanol and freezing at − 20 °C) on DNA quality parameters such as DNA concentration (ng/mL), purity (absorbance ratios 260/280 and 260/230), and integrity (DNA integrity number, DIN, a numerical assessment of DNA fragmentation) across different decomposition condition categories (DCC) in stranded small cetaceans. Additionally, we examined the interplay of these variables with storage time, and their impact on final DNA quality outcomes. This study aims to advance knowledge on sampling and preservation protocols that can be applied to stranding networks worldwide, particularly those with limited financial resources, to facilitate the collection of high-quality samples for genetic analysis in small cetaceans.

## Results

### Influence of tissue matrix, decomposition condition category (DCC), preservation method and storage time on DNA quality measures

The influence of the analyzed variables on DNA concentration (ng/µL) differed between both measuring instruments (i.e. NanoDrop 1000 Spectrophotometer and Agilent TapeStation system).

Measurements using the NanoDrop (ND) revealed that the tissue matrix (*p* < 0.001), DCC (*p* < 0.001), and storage time (*p* = 0.012) significantly affected concentration levels. For the TapeStation (TS), significant differences were observed for the tissue matrix (*p* < 0.001), DCC (*p* < 0.001), and the interaction between the tissue matrix and DCC (*p* = 0.046).

**Fig. 1:**
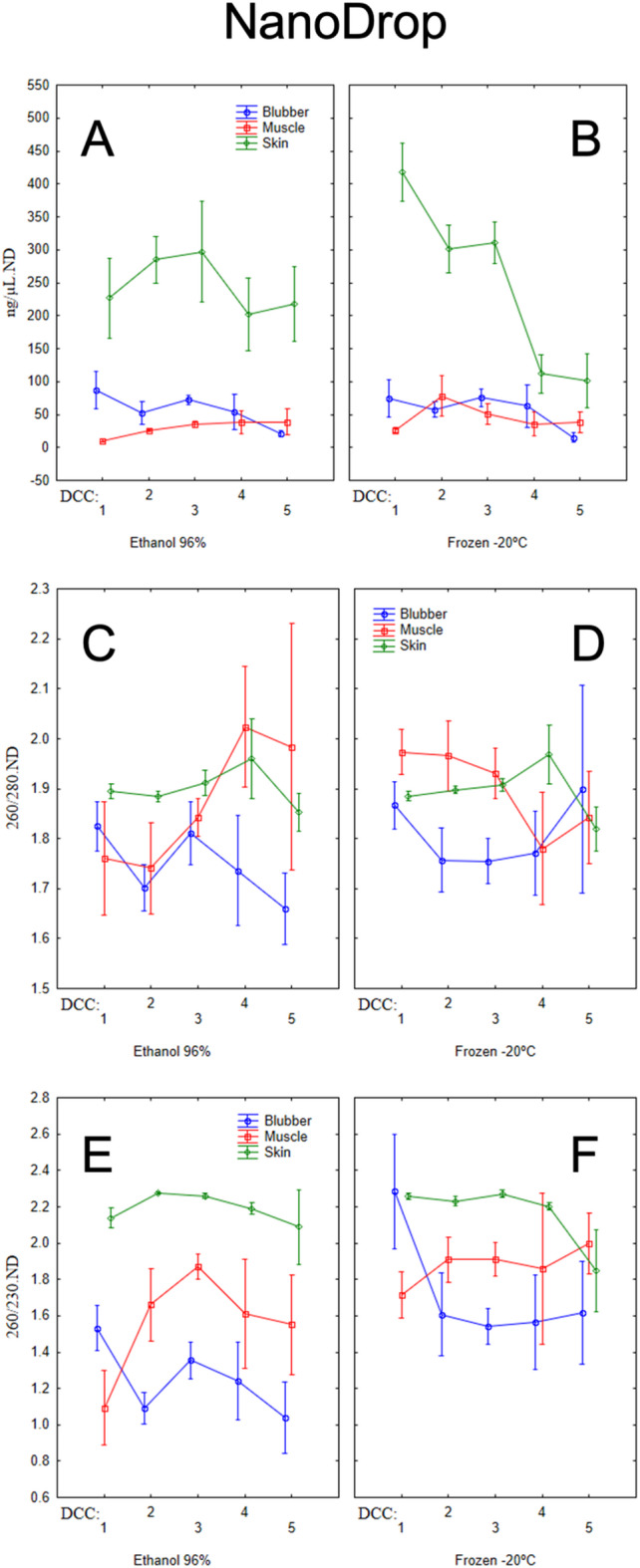
DNA concentration (**A** & **B**), 260/280 ratio (**C** & **D**), and 260/230 ratio (**E** & **F**) of different tissue matrices (blubber, muscle, and skin) preserved in 96% ethanol (**A**, **C** & **E**) or frozen at − 20 °C (**B**, **D** & **F**), measured using a NanoDrop spectrophotometer.

The differences observed between tissue matrices were mostly attributable to DNA yields from skin samples, which had higher concentration values than muscle and blubber samples. Overall, higher DNA concentration values were also observed in earlier DCCs (1 to 3, corresponding to fresher carcasses) compared to advanced stages of decomposition (4 and 5), with a more pronounced decay in skin samples (Fig. 1A-B, and 2A-B). Samples with longer storage times also displayed significantly higher DNA concentrations.

**Fig. 2:**
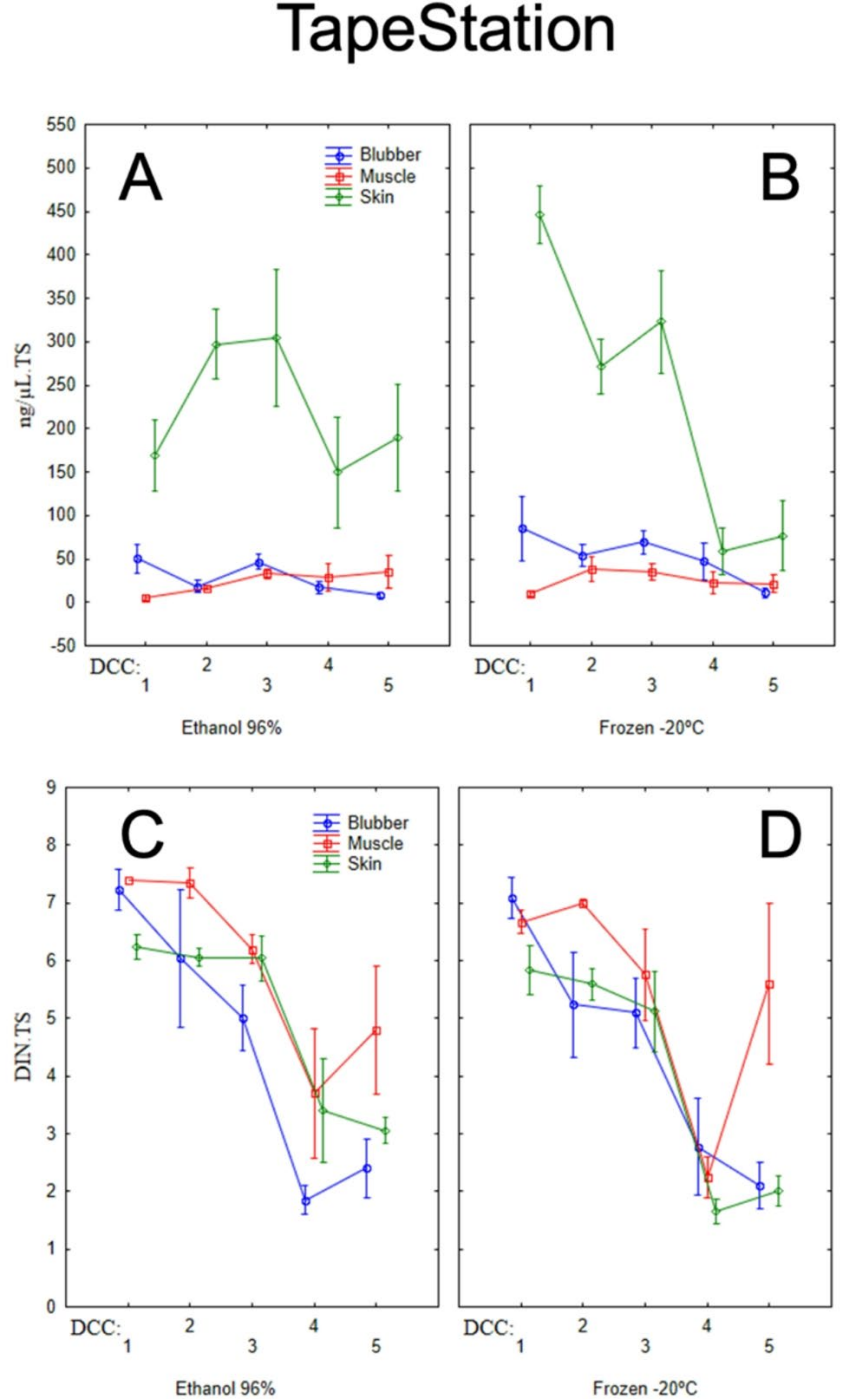
DNA concentration (**A** & **B**), and DNA Integrity Number (**C** & **D**) of different tissue matrices (blubber, muscle, and skin) preserved in 96% ethanol (**A** & **C**) or frozen at − 20 °C (**B** & **D**), measured using a TapeStation system.

Absorbance at 280 nm (260/280) was significantly affected by the tissue matrix (*p* < 0.001) and storage time (*p* = 0.046). For absorbance at 230 nm (260/230), measurements revealed a significant effect associated with the preservation method (*p* < 0.001), the tissue matrix (*p* < 0.001), and the interaction between the preservation method and the tissue matrix (*p* = 0.013).

Each tissue matrix exhibited their own absorbance profile for both absorbance ratios, depending on the preservation method (Fig. 1C – F). Additionally, a significant increase in absorbance values at 280 nm was observed with prolonged storage time.

DNA integrity was significantly influenced by DCC (*p* < 0.001), tissue matrix (*p* < 0.001), storage time (*p* = 0.025), and preservation method (*p* = 0.032).

Higher DNA integrity values were generally observed in fresher samples (DCC 1–3), with a sharp decline corresponding to DNA fragmentation in later stages of decomposition (DCC 4 and 5). However, an exception to this trend was observed in muscle samples from mummified carcasses (DCC 5) frozen at -20 °C, which exhibited high DNA integrity values relative to the preceding DCC level 4 (Fig. 2C – D).

For advanced decomposition categories (DCC 4 and 5), ethanol (96%) resulted in higher DNA integrity values for skin samples, while for muscle samples, frozen storage led to higher DNA integrity values in mummified carcasses (Fig. 2C – D). Additionally, DNA integrity values increased significantly with storage time.

Discriminated values of DNA concentration, absorbance ratios, DIN, and storage time for each analyzed sample are shown in Supplementary Table 1. Raw statistical values are available in Supplementary File 1.

### DNA extraction variability analysis

Replicated DNA extraction revealed significant differences in DNA concentration (ng/µL) between initial extraction and replicate groups for DCC (*p* < 0.001) and tissue matrix (*p* < 0.001), but not for the preservation method (*p* = 0.081) or for the interaction between variables.

For the absorbance ratios, significant differences were observed at 280 nm and 230 nm, particularly in the interactions between replicates and DCC, preservation method, and tissue matrix (*p* < 0.001).

While for absorbance at 280 nm, differences between the initial extraction and replicates were observed only for muscle samples (DCC 4 preserved in ethanol 96% and DCC 5 preserved frozen), for absorbance at 230 nm, differences were observed across multiple DCCs, preservation methods, and tissue matrices (Supplementary Table 2). Raw statistical values are presented in Supplementary File 2.

### Correlation

**Fig. 3:**
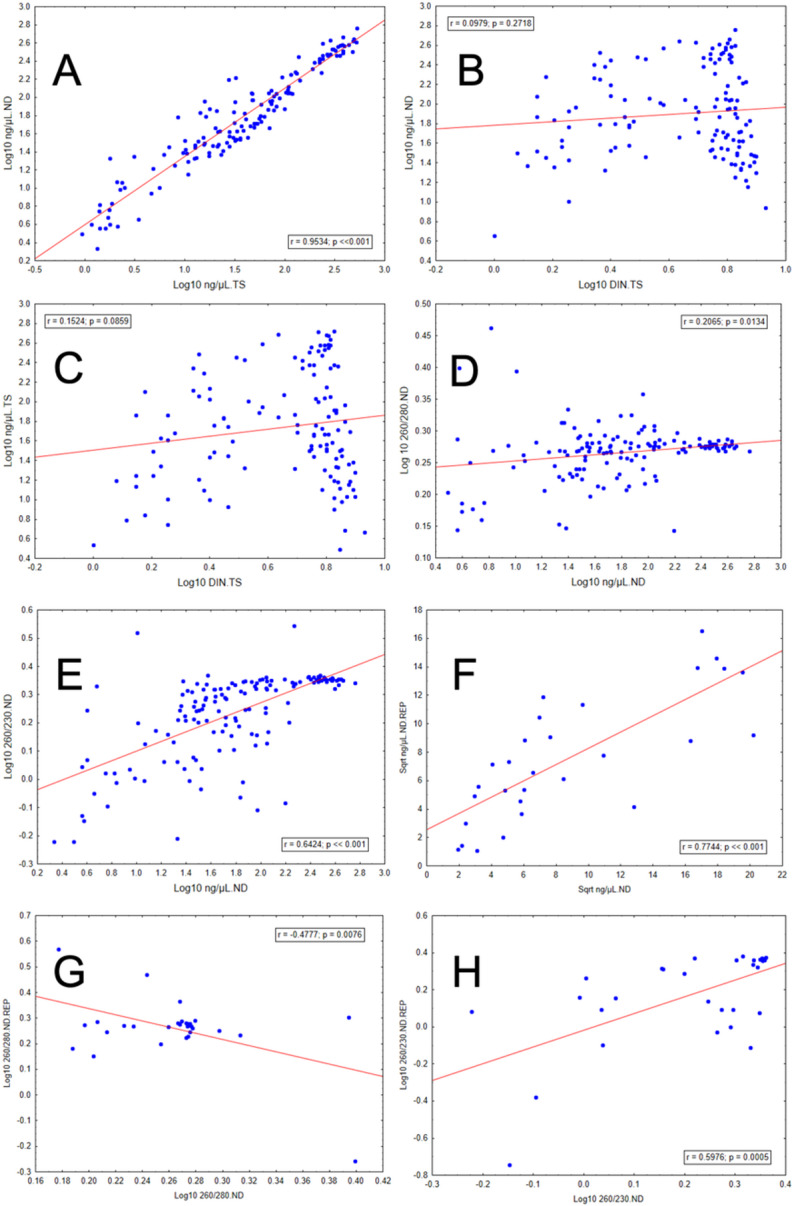
Linear correlations between: (**A**) DNA concentration measured with the NanoDrop spectrophotometer and the TapeStation system; (**B**) DNA concentration measured with the NanoDrop spectrophotometer and the DNA Integrity Number; (**C**) DNA concentration measured with the TapeStation system and the DNA Integrity Number; (**D**) 260/280 absorbance ratio and DNA concentration measured with the NanoDrop spectrophotometer; (**E**) 260/230 absorbance ratio and DNA concentration measured with the NanoDrop spectrophotometer; (**F**) DNA concentration from repeated extraction and from the initial extraction; (**G**) 260/280 absorbance ratio from repeated extraction and from the initial extraction; (**H**) 260/230 absorbance ratio from repeated extraction and from the initial extraction.

A highly significant positive correlation was found between the DNA concentrations (ng/µL) obtained from ND and TS (*r* = 0.953, *p* < 0.001, Fig. 3A).

Similarly, a significant positive correlation was found between absorbance at 280 nm (260/280) and DNA concentration, although r values are relatively low (*r* = 0.2065, *p* = 0.013, Fig. 3D). For absorbance at 230 nm (260/230), a significant positive correlation was observed with DNA concentration (*r* = 0.6424, *p* < 0.001, Fig. 3E).

A highly significant positive correlation was found between the DNA concentration (ng/µL) obtained from the original extraction group and the replicate group (*r* = 0.7744, *p* < 0.001, Fig. 3F). Regarding absorbance ratios, significant negative and positive linear relationships were observed for absorbance at 280 nm (*r* = -0.4777, *p* = 0.008, Fig. 3G) and 230 nm (*r* = 0.5976, *p* < 0.001, Fig. 3H), respectively.

No significant linear relationships were found between DNA integrity (DIN) and DNA concentration (ng/µL) obtained from ND (*r* = 0.0979, *p* = 0.272, Fig. 3B) or TS (*r* = 0.1524, *p* = 0.086, Fig. 3C).

### General discriminant analysis

According to the different sequencing quality control criteria, several combinations of tissue matrix and preservation method scored significantly higher on the DNA characteristics criteria (Supplementary Table 3). Based on quality control pass rates, a table was generated showing the highest-scoring tissue matrix and preservation method combination for each DCC and sequencing technique (Table 1). For those DCC and sequencing techniques where no tissue matrix, preservation method, or their combination scored significantly higher, no differences were assumed between the available options.

**Table 1:** Highest-scoring tissue matrix (in bold) and preservation method (in brackets) for sequencing quality control criteria, shown by decomposition condition category (DCC), evaluation criteria (DNA concentration, purity, and DIN), and sequencing technique. Abbreviations: **B** – blubber, **M** – muscle, **S** – skin, (Frozen) – frozen at − 20 °C, (Ethanol) – 96% ethanol, = – no difference between tissue matrices, (=) – no difference between preservation methods, All Fail – no sample passed the sequencing quality control criteria, ND – sequencing quality control criteria not determined.

DCC	1			2			3			4			5		
Criteria	Concentration	Purity	DIN	Concentration	Purity	DIN	Concentration	Purity	DIN	Concentration	Purity	DIN	Concentration	Purity	DIN
Sanger Sequencing	**B** (=) **M** (Frozen) **S** (=)	**B** (Frozen) **S** (Ethanol)	**B** (=)	**=** (=)	**S** (=)	**M** (=)	**=** (=)	**M** (=) **S** (=)	**=** (=)	**=** (=)	**=** (=)	All Fail	**S** (=)	**S** (=)	**=** (=)
WGS (non-FFPE)	**B** (=) **S** (=)	**B** (Frozen) **S** (Ethanol)	**B** (=)	**=** (=)	**S** (=)	**M** (=)	**=** (=)	**M** (=) **S** (=)	**=** (=)	**S** (=)	**=** (=)	All Fail	**=** (=)	**S** (=)	**=** (=)
WGS (FFPE)	**B** (=) **S** (=)	ND	**=** (=)	**=** (=)	ND	**=** (=)	**=** (=)	ND	**=** (=)	S (=)	ND	**=** (=)	**=** (=)	ND	M (=)
PCR-free WGS	**B** (Frozen) **S** (=)	**B** (Frozen) **S** (Ethanol)	**=** (=)	**S** (Frozen)	**S** (=)	**=** (=)	**=** (=)	**M** (=) **S** (=)	**=** (=)	S (=)	**=** (=)	**=** (=)	**S** (=)	**S** (Frozen)	**M** (=)
Nextera DNA XT	**=** (=)	**B** (Frozen) **S** (Ethanol)	**B** (=)	**=** (=)	**S** (=)	**M** (=)	**=** (=)	**M** (=) **S** (=)	**=** (=)	**=** (=)	**=** (=)	All Fail	**=** (=)	**S** (Frozen)	**=** (=)
PacBio CLR	**S** (=)	**S** (Frozen)	**=** (=)	**S** (Frozen)	**M** (Frozen) **S** (=)	All Fail	**S** (=)	**M** (=) **S** (=)	All Fail	**S** (=)	**B** (Frozen) **M** (Ethanol) **S** (Ethanol)	All Fail	**S** (=)	**S** (=)	All Fail
PacBio HiFi	**S** (=)	**=** (=)	**=** (=)	**S** (Frozen)	**M** (Frozen) **S** (=)	All Fail	**S** (=)	**M** (=) **S** (=)	All Fail	**S** (=)	**S** (=)	All Fail	**S** (=)	**=** (=)	All Fail
Nanopore Promethion	**S** (=)	**=** (=)	**B** (=)	**S** (=)	**M** (Frozen) **S** (=)	**M** (=)	**S** (=)	**M** (=) **S** (=)	**=** (=)	S (=)	**B** (Frozen) **M** (Ethanol) **S** (Ethanol)	All Fail	**S** (=)	**S** (=)	**=** (=)
Target Capture (Genomic)	**=** (=)	**B** (Frozen) **S** (Ethanol)	**B** (=)	**=** (=)	**S** (=)	**M** (=)	**=** (=)	**M** (=) **S** (=)	**=** (=)	**=** (=)	**=** (=)	All Fail	**=** (=)	**M** (Frozen)	**=** (=)
Target Capture (FFPE)	**=** (=)	ND	**=** (=)	**=** (=)	ND	**=** (=)	**=** (=)	ND	**=** (=)	**=** (=)	ND	**=** (=)	**S** (=)	ND	**M** (=)
Methylation (WGBS)	**B** (=) **S** (=)	**=** (=)	ND	**=** (=)	**S** (=)	ND	**=** (=)	**S** (=)	ND	**S** (=)	**=** (=)	ND	**=** (=)	**=** (=)	ND
Methylation (RRBS)	**B** (Frozen) **S** (=)	**=** (=)	ND	**S** (Frozen)	**S** (=)	ND	**=** (=)	**S** (=)	ND	**S** (=)	**=** (=)	ND	**S** (=)	**=** (=)	ND

## Discussion

A systematic comparison of the quality of DNA obtained from different sampling strategies (i.e., tissue selection and preservation method used) from dead stranded cetaceans for downstream genetics analyses has been lacking. Although different guidelines have been established, they were not supported by a calibration of standard quality control measures for specific tissue matrices to be sampled and the preservation methods to be employed. In this study, different properties of the analyzed tissues (i.e., muscle, blubber, and skin)—in terms of DNA concentration, purity, and integrity—were assessed across two commonly used preservation methods (96% ethanol and frozen at − 20 °C) and decomposition condition categories (i.e., 1 to 5, from fresh to advanced decomposed carcasses).

Overall, skin consistently yielded the highest DNA concentrations, whereas muscle showed higher DNA integrity and less degradation in carcasses at advanced stages of decomposition, despite producing lower concentrations. The study also revealed significant variation in DNA concentration across DCCs, with a particularly strong decline from DCC 3 to 4 in frozen skin samples. Together with a lack of correlation between concentration measures and DNA integrity, the current study therefore shows that tissue choice is an important consideration depending on the intended application of the extracted DNA. These findings provide valuable insights into the suitability of different tissue matrices for genetic analysis under varying conditions, such as preservation method and decomposition stage. The results highlight the need to adjust sampling protocols according to the decomposition condition category and the best suitable preservation method, to maximize the usability of the material collected for downstream genetic applications.

### DNA concentration

For genetic applications where DNA quantity is a priority, the present results show that skin consistently yields higher DNA concentrations than muscle and blubber in cetaceans, as measured by both NanoDrop and TapeStation measurements. Importantly, despite the differences in concentration values obtained with the two measurement methods (Supplementary Material 1), which is expected given their differences in reproducibility, a highly significant correlation was observed between them. This finding supports the use of either method for DNA concentration estimation. The higher DNA yield from skin can facilitate extraction and help ensure that sufficient material is available for genetic analyses^22–24^. In contrast, DNA concentrations from muscle and blubber were generally much lower across all five DCCs, ranging from nearly undetectable levels to 100 ng/µL. This disparity also suggests a practical advantage in using skin tissue for genetic analyses in cetacean research based on stranding material, because it provides consistently high DNA yield in both fresh and advanced decomposed specimens. Furthermore, the high DNA yield obtained from skin supports a wide range of genetic applications, including population genetics, phylogenetics, genetic diversity, adaptation, and evolutionary studies in cetaceans (as shown in Table 1).

This tissue-specific difference in DNA concentration has also been reported in humans and other animals, with skin yielding higher DNA concentrations compared to muscle and adipose tissue (which produces the lowest yields)^25–27^. These differences may result from histological structure, interference from tissue components, and tissue resilience. Mammal skin typically contains a high number of nucleated cells, such as keratinocytes and fibroblasts^28^, although keratinocytes at the outer skin layers tend to lose their nucleus. Odontocete skin is thicker compared to other mammals but structurally less complex, contains high densities of active keratinocytes that more often retain their nucleus, as well as melanocytes, Langerhans cells and Merkel cells, which are all nucleated^29–31^. In contrast, adipose tissue, is primarily composed of adipocytes, proportionally large cells whose content consists mainly of a large lipid droplet with proportionally smaller nuclear material^32^. Cetacean blubber is a specialized type of adipose tissue, composed of three structurally distinct layers, with the middle layer composed of lower number of relatively larger adipocytes. The other two layers, however, contain higher proportion of fibrous material, with the deeper layer being the most metabolically active^33–35^. Depending on the metabolism state of individual animals, this layer could contain a higher density of nucleated cells such as fibroblasts and erythrocytes. This could explain why DNA yields from blubber were closer to those obtained from muscle (and in some cases higher), than has been observed in other terrestrial mammals.

Alternatively, the high lipid content may also cause contamination during cell lysis and DNA purification using column-based methods, thereby reducing overall DNA yield^26^. In this study, we did not carry any step aimed at removing the lipid content of the tissue, and cannot exclude the possibility that doing so would increase DNA yields from blubber. Similarly, muscle tissue is rich in structural proteins, such as myosin and actin, which can hinder enzymatic lysis^36^. Moreover, muscle has been shown to undergo faster decomposition compared to other tissues, such as skin^37^. For these reasons, skin can be expected to provide higher DNA concentrations relative to other tissue matrices. Its superficial location facilitates easier collection, making it particularly practical under field conditions.

This study also shows that DNA concentration is influenced by decomposition stage and preservation method. Samples stored at − 20 °C showed the highest DNA concentrations in DCCs 1, 2, and 3, with a gradual decrease in DCCs 4 and 5, while samples preserved in ethanol showed relatively stable DNA concentrations across all DCCs. The higher concentrations observed in frozen samples from DCCs 1, 2, and 3 may indicate that freezing is more effective at preserving the higher DNA concentrations typical of early decomposition stages, a property widely recognized for this preservation method. The rate and extent of DNA fragmentation are influenced by multiple factors, including enzyme activity, temperature, pH, and ion concentrations. Conditions that preserve tissues, such as low temperatures, can slow or halt nuclease activity, maintaining longer DNA fragments suitable for analysis. Therefore, higher DNA concentrations are expected in samples where decomposition has not yet progressed extensively (i.e., DCCs 1–3). The decrease in DNA concentration observed in the advanced decomposition categories is particularly pronounced in skin samples. In contrast, DNA concentrations in muscle and blubber appear relatively stable across the decomposition spectrum. These results may be explained by the influence of environmental factors on tissue integrity. After death, tissues undergo substantial changes that affect DNA stability. The cessation of oxygen and nutrient supply reduces ATP production, forcing cells into anaerobic metabolism which leads to the accumulation of acidic by-products, particularly affecting cells with high energy demands. Depending on the circumstances, cells may undergo apoptosis—a regulated process in which DNA is cleaved into predictable fragments—or necrosis, an uncontrolled breakdown resulting in random DNA degradation. DNA continues to degrade postmortem due to both endogenous and exogenous nucleases. Furthermore, the release of nutrient-rich fluids after death promotes microbial proliferation, which further accelerates DNA degradation^38^.

Among the studied tissues, skin is the most exposed to external conditions such as sunlight, rain, temperature, moisture, scavenging, external microbial activity and carcass dragging^39,40^. Consequently, in carcasses at advanced decomposition stages that have been exposed to these factors for longer periods, skin integrity is more severely affected than that of other tissues. By contrast, muscle and blubber are afforded a degree of protection by the overlaying skin, reducing the impact of external drivers on their DNA yield. Therefore, when collecting skin samples for genetic analysis, considerations regarding skin integrity are essential. Sampling should focus on areas that have been less affected by environmental factors, such as by avoiding regions with high exposure to direct sunlight.

However, our results also showed no correlation between concentration readings from either the NanoDrop or the TapeStation, and levels of DNA fragmentation as measured by the DIN quantification. DNA integrity is influenced by tissue degradation, damage, and contamination, whereas DNA concentration depends on factors such as sample preparation, extraction efficiency, and measurement methods. This is an important result to consider, because it suggests that high DNA concentrations do not in themselves guarantee successful genetic analyses, since effective amplification for certain genetic applications - e.g. genomics—requires high-integrity DNA (see Table 1).

### DNA purity

The readings at the absorbance of 280 nm varied significantly across the matrices studied, however, and as discussed further below, these values are likely to be less critical for downstream applications. Skin was the most consistent tissue across both preservation methods, consistently scoring ratios above 1.8. Interestingly, this pattern was generally maintained across decomposition stages, highlighting low DNA contamination in this matrix. Typically, pure DNA presents a 260/280 ratio of ~ 1.8^41^, and in common laboratory practice, DNA samples with 260/280 ratios above 1.8 are considered pure and suitable for use in most downstream applications^42^.

Blubber, on the other hand, generally presented ratios below 1.8, with samples preserved in 96% ethanol falling below the acceptable range after DCC 3. Muscle showed different trends depending on the preservation method. In 96% ethanol, lower ratio values were observed in fresher decomposition stages. Notably, muscle tissue exhibited an unexpected increase in absorbance after the first two DCCs, remaining within the acceptable range (up to 2.0). In frozen samples, the opposite trend was observed, with more stable and acceptable ratios in fresher samples compared with more decomposed carcasses.

Changes in the 260/280 ratio may indicate protein contamination when values are lower than expected, or RNA contamination when values are higher^42^. However, the role of DNA concentration in the sample must also be considered. In low-concentration samples (i.e., < 20 ng/µL), the reliability of purity ratios decreases significantly, with measurements showing high variability. This variability, although less pronounced, is still observed at higher DNA concentration ranges (i.e., 20–50 ng/µL)^42^. In the current study, both blubber and muscle consistently showed DNA concentrations below 100 ng/µL, in contrast with skin samples. This factor may partially explain the more variable purity ratio trends observed in blubber and muscle. Interference of residual reagents associated with the extraction protocol can also explain such variation^43^.

Alternatively, higher 260/280 ratios in muscle samples from decomposed carcasses preserved in 96% ethanol and from fresh frozen carcasses could reflect RNA contamination. However, contaminations below 15% do not significantly increase the 260/280 ratio and are difficult to detect with microvolume spectrophotometers^42^.

The absorbance measured at 230 nm also varied significantly among the matrices, with more pronounced differences than those observed at 280 nm. As with the 260/280 ratio, skin exhibits the most consistent purity ratios across both preservation methods and decomposition stages, confirming its low level of DNA contamination.

In general, blubber and muscle showed lower ratio values than skin. Differences between preservation methods are also evident, with both blubber and muscle matrices kept in 96% ethanol falling below the reference range (i.e., 1.8–2.2, see below) for most samples. In the − 20 °C preservation method, muscle samples were generally within the reference range, whereas blubber samples remained below, highlighting the role of freezing in preserving DNA quality in muscle.

For the 260/230 ratio, DNA is generally considered pure if within the range of 1.8–2.2^41^, although some authors propose narrower ranges such as 2.0–2.2^43^. Several contaminants can interfere with the 260/230 purity ratio, to which it is particularly sensitive, resulting in lower values as observed in some muscle and blubber samples. The low ratios may be partially attributed to contamination with residual chaotropic salts such as guanidine thiocyanate and guanidine hydrochloride, EDTA, non-ionic detergents, ethanol, and/or proteins^42^. Extraction kits often employ guanidine salts such as guanidine hydrochloride or guanidine thiocyanate in binding buffers^43,44^. Non-ionic detergents are commonly included in lysis buffers, and EDTA is frequently used in elution buffers^42^. These contaminants absorb strongly at 230 nm, substantially reducing the 260/230 ratio and complicating efforts to achieve the desired purity for downstream applications^45^. However, 260/230 ratios typically show higher variability than 260/280 ratios, and in many cases the impact of such contaminants on downstream applications is negligible^42^. As with the 260/280 ratio, the 260/230 ratio is also sensitive to low DNA concentrations^42^, which may also explain the observed variation in muscle and blubber.

### DNA integrity

Differences in DNA integrity number (DIN) of the genetic material were observed across matrices and DCC stages. Notably, muscle tissue showed the highest DIN values compared with skin and blubber under both preservation methods, except specifically at DCCs 1 and 4 under the − 20 °C preservation method. Early DCC stages exhibited higher DNA integrity values, which generally declined as DCC progressed. As discussed above for DNA concentration, this is expected if in fresher tissues enzymatic degradation had not significantly advanced^16^. DIN values dropped sharply after DCC 3 across all matrices, suggesting that samples from specimens at DCC stages later than 3 should prioritize matrices with higher DNA integrity, such as muscle. After DCC 4, however, all matrices tended to stabilize or even increase their DIN values. Muscle tissue exhibited a substantial increase compared to other tissues, particularly in frozen samples. This result was unexpected, as DCC 5 represents the most advanced stage of degradation, and based on the principle of higher enzymatic degradation with more advanced composition, it would be expected that muscle would follow the overall trend of decreasing values from DCC 1 to DCC 5.

A protective effect from tissue organization (i.e., stratification with skin as the most external layer, blubber as the middle section, and muscle as the most internal layer) may explain why carcasses in advanced decomposition showed muscle with higher DNA integrity values compared with skin and blubber, which were more severely exposed to environmental effects. Prolonged exposure to external factors such as UV radiation, temperature fluctuations, and mechanical trauma in the outer tissues’ layers, can lead to higher tissue decomposition and, consequently, DNA degradation^14,38^. On the other hand, mummification can also play a key role in the dynamics observed. The mummification process leads to desiccation of the soft tissues, which slows DNA decay pathways such as depurination^46,47^, and suppresses microbial and enzymatic activity^48^. In this sense, samples derived from mummified carcasses, as opposed to those in advanced stages of decomposition, may have the potential to yield higher-quality DNA.

Notably, both DIN and DNA concentration were shown to increase significantly with storage time, which is a somewhat counterintuitive result. Regarding DNA concentration, the observed trend could be explained by continued chemical and enzymatic degradation of tissue during storage, particularly through the action of nucleases^49^. Such degradation likely disrupts cell structure, facilitating DNA release during extraction and resulting in higher yields. DIN values could instead be explained by sampling bias: samples from carcasses in fresher DCCs (i.e., 1 and 2) were less readily available in the sample archive than those from animals in advanced decomposition stages. As a result, early-stage samples from tissue banks might end up having higher storage times due to their rarity, while advanced decomposition samples were more readily obtained from stranding cases happening during the sampling process for this study. For this reason, no recommendation on storage time is provided within the scope of this study.

Although DNA integrity was assessed in this study using the TapeStation system to obtain numerical values for more robust statistical comparisons, it is important to tailor DNA quality assessment methods to the financial capacity of each research institution. Alternatively, other approaches may provide suitable estimates of DNA integrity at more accessible costs (e.g., agarose gel electrophoresis).

### Sampling guidelines

In the context of genetic analysis of small cetaceans, sampling guidelines such as those proposed in this study can benefit two main groups: researchers conducting genetic analyses and stranding networks that collect and curate tissue banks for future use. Although our study did not directly tests differences in the quality of downstream generated data (e.g. PCR or genomic library preparation), for researchers, who typically understand the sequencing techniques to be applied, the detailed sampling guideline proposed here (Table 1) can serve as a reference for selecting samples most likely to perform well in terms of DNA characteristics under different sequencing methodologies and decomposition conditions.

**Fig. 4:**
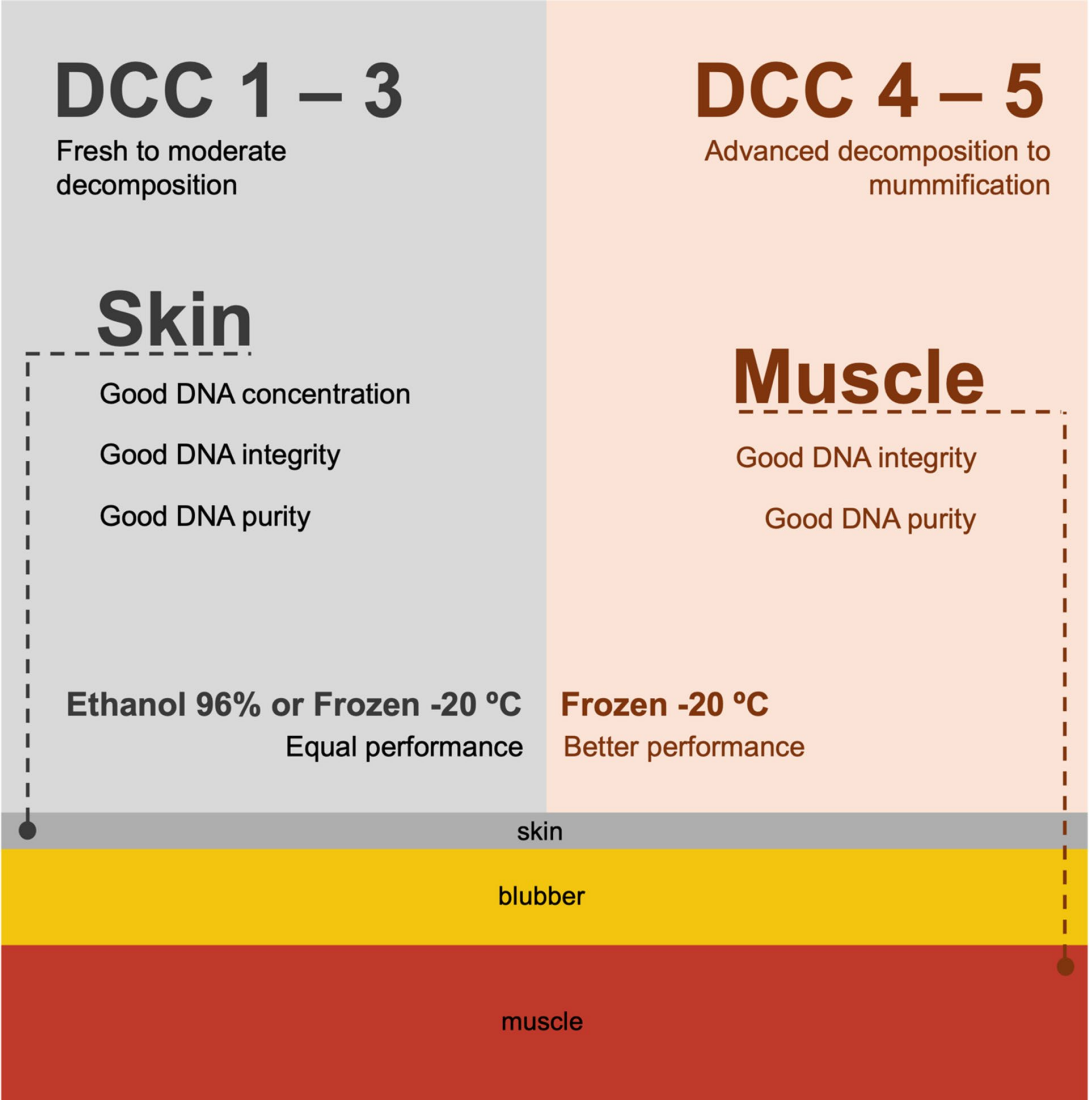
Simplified sampling guidelines.

For stranding networks seeking to establish best practice guidelines for the sampling and storage of tissues for genetic analysis, a more user-friendly and simplified guideline is recommended (Fig. 4). This guideline, derived from the results and conclusions of the multivariable analysis presented earlier, is intended as a general framework for sampling. It is not meant to replace practices tailored to the specific needs of individual stranding response organizations or research teams. Despite the guidelines provided in this study regarding tissue type and preservation method, it is important to note that researchers should consider other available preservation options and tissue matrices, and evaluate which are best suited to their specific research needs and practical context.

Overall, our results suggest that skin is the preferred tissue for applications that require maximum DNA yield, while muscle is the preferred tissue for applications that require the DNA with least degradation levels. Therefore, stranding networks are advised to sample both skin and muscle samples from each carcass. For high decomposition levels, the recommendation is that the muscle tissue should be obtained from deeper layers of the body surface whenever possible. It is important to note, however, that those two tissues require different processing protocols during extraction. Skin required more careful tissue disruption prior to Proteinase K digestion, required more lengthy digestion times, and always resulted in more noticeable undigested tissue residue. In this study, the amount of tissue used between matrices was standardized, but higher DNA concentration could potentially be obtained by use of larger starting material. Therefore, the lower concentration of muscle, given its faster processing time and lower intact residue, could be compensated by using larger starting material (although not tested in this study).

Furthermore, replicate analyses showed that the DNA extraction procedure carries significant variability in terms of DNA concentration. Variability among extraction methods, including column-based approaches, has been reported previously^50,51^. Technical variability can also occur between extraction replicates^52^. Such variability may arise from differences in the biological composition of the sample as well as from the extraction and quantification procedures themselves^53^. In the present study, factors such as different operators and variation in extraction and measurement days likely contributed to the observed differences, but higher concentration consistency was obtained in extractions from skin. Importantly, although variability in DNA concentration was detected, the relationship between initial and replicate DNA concentration results was highly significant, supporting consistency in the methodology. Therefore, it is recommended that stranding networks aim at sampling larger muscle samples relative to skin, to account for the need of processing more material for each DNA extraction and the need for more replicates per sample.

Although current results suggest blubber to be the least preferred tissue for DNA extraction, it is emphasized that it can still provide DNA yields comparable to muscle with DIN integrity comparable to skin. These results could have been improved using extraction methods that more effectively remove the lipidic content of the tissue (e.g. phenol-chloroform, although it is more labor-intensive). Therefore, our results do show that blubber can still be used for DNA extraction if not enough muscle or skin is available from the same specimen. Therefore, if blubber is collected for some other purpose, it could still be a viable source of DNA from long term archives.

Regarding DNA purity, from a practical perspective, elevated 260/280 ratios are not considered problematic^42^, and such samples remain suitable for downstream applications. This consideration is particularly relevant given that storage time was shown in the present study to significantly increase this ratio. Therefore, although differences in the purity levels obtained from the samples were found, it is suggested that for long term storage purposes, focusing on this parameter during the sampling stage is not considered a priority.

Finally, the results demonstrate a strong correlation between DNA concentration measurements obtained with the TapeStation and NanoDrop, indicating that both methods provide consistent and comparable estimates of DNA quantity. The NanoDrop is user-friendly, requires only a small sample volume, delivers rapid measurements of DNA concentration and purity, and is less costly than methods such as the TapeStation. Both techniques enable the extrapolation of results from one method to the other, a strategy that may be particularly useful in research projects where only one approach was applied but inference about the other method is valuable. Contrastingly, DNA concentrations did not correlate with DNA integrity, meaning that high DNA concentration is not a reliable proxy for DNA integrity. Therefore, if DNA integrity is an important consideration, it is recommended that researchers test for this specific quantity before proceeding with their analyses.

## Conclusion

In conclusion, this study provides a first comparison of sampling strategies to maximise DNA quality for genetic analysis from stranded cetaceans, addressing a previous knowledge gap. The DNA concentration, purity, and integrity of different tissues (muscle, blubber, and skin) under two commonly used preservation methods (ethanol 96% and frozen − 20 °C) and five decomposition stages (i.e., from fresh to mummification) were analyzed. This study emphasizes the necessity of adapting sampling protocols to the specific decomposition stage and suitable preservation method. Selecting the appropriate tissue matrix and preservation method based on the decomposition state is essential for optimizing DNA samples for further analyses. These insights inform best practices for cetacean genetic analysis, ensuring effective use of DNA samples. Accurate DNA quantification is important for reliable genetic data, particularly for techniques requiring high-quality DNA. Proposing these sampling guidelines aims at standardizing cetacean genetic research, enhancing the quality and consistency of data across studies.

## Methods

### Sample collection

Tissue samples were collected from delphinid carcasses stranded along the west coast of Portugal between Lourinhã and Setúbal, with different decomposition condition categories (DCC). Specimen sourcing was performed by the Lisbon and Tagus Valley Stranding Network (RALVT), and sampling was conducted within the scope of regular necropsy investigations. Sample collection was performed under licenses granted by the competent authority - Instituto da Conservação da Natureza e das Florestas, IP (Licenses Nos. 170/2022/CAPT, 883/2022/CAPT, 462/2023/CAPT, 463/2023/CAPT, 464/2023/CAPT, and 465/2023/CAPT).

Samples were collected between December 2022 and November 2023. Three different tissues were sampled for each specimen: skin, blubber, and muscle. Tissues were sampled on a vertical axis (from surface to internal tissues) systematically in the same body area in all animals (caudoventral to the dorsal fin, adjacent to the epaxial musculature region). Collection was carried out using sterile equipment (i.e. scalpel blades and forceps), which were replaced (scalpel blades) or disinfected with hypochlorite 10% (forceps) between sampling to avoid cross-contamination between specimens. All samples were collected and stored for a period of no longer than 13 months before DNA extraction. Each sample was stored using two distinct preservation methods: frozen at -20 °C in 0.4 L resealable plastic bags (IKEA, Delft, Netherlands) and kept at room temperature in 120 mL sterile plastic containers (FL Medical, Torreglia, Italy) with ethanol 96% (AGA, Prior Velho, Portugal). Preservation method selection followed these criteria: being referenced as suitable for genetic material storage in international sampling guidelines for small cetaceans^9,17^; being readily accessible to stranding networks with varying financial capacities; and reflecting the authors’ personal preferences regarding preservation methods for genetic analyses.

### Tissue bank assemblage

The final experimental sample scheme was composed of a combination of three tissue matrices in two preservation methods over five DCC, namely: 1 – extremely fresh carcass, just dead; 2 – fresh carcass; 3 – moderate decomposition; 4 – advanced decomposition; 5 – mummified or skeletal remains. Each DCC group was represented by five specimens (*n* = 25). Each tissue matrix (blubber, muscle, and skin) was sampled in duplicate for each specimen, and samples were evenly distributed between the two preservation methods (frozen − 20 °C and ethanol 96%). The selection of DCC to be included in the study, as well as the diagnostic criteria to assign specimens to each category, were based on established guidelines^9^. The rationale for guideline selection included expert-group-produced guidelines commonly used by stranding networks internationally. Storage time (in days) was recorded for each sample and corresponded to the time gap between necropsy date and the mean time in which DNA extraction occurred. The exact DNA extraction dates for individual samples were not recorded, but all extractions were performed within a three-week period.

All animals included in this study were short-beaked common dolphins (*Delphinus delphis*), except for two striped dolphins (*Stenella coeruleoalba*) specimens included in the DCC 1 group. This species diversity reflects the stranding trends in the sampled region. Sample collection was achieved for almost all intended stratifications, except for two muscle samples stored in ethanol 96% in the group DCC 1, one muscle sample stored at -20 °C in the group DCC 1, and one muscle sample stored at -20 °C in the group DCC 2, which are missing from the final dataset.

### Genomic DNA extraction

Genomic DNA was extracted from all samples collected using the NucleoSpin Tissue kit (Macherey-Nagel, Düren, Germany). DNA extraction was performed following the manufacturer’s standard protocol for animal tissue, with minor adaptations. Tissue samples were cut into small pieces on a Petri dish disinfected with hypochloryte 10% using a sterile scalpel, to increase the surface area and facilitate the lysis process. Incubation with lysis buffer and proteinase K was performed until the tissue was fully digested, which, for the muscle and blubber samples, occurred after 1.5 h. The skin tissue, however, required overnight incubation to maximize tissue breakdown, which never fully occurred. After the extraction procedure, DNA extraction products were stored in 1.5 mL sterile tubes (Ratiolab GmbH, Dreieich, Germany) at 4 °C until further processing.

To assess the variability associated with the extraction procedure, replicates were performed, including one representative of each combination under study (DCC, tissue matrix, and preservation method), for a total of 30 samples.

### DNA concentration, purity and integrity assessment

Yield and purity of the extractions were analyzed using a NanoDrop 1000 Spectrophotometer (Thermo Fisher Scientific, Waltham, USA), with the supplied software (NanoDrop 1000 version 3.7.1) through the “Nucleic Acid” application module. For each sample, the two absorbance ratios (260/280 and 260/230) as well as the DNA concentration (ng/µL) were recorded.

Before each sample absorbance reading, the equipment was blanked using 2 µL of distilled water. A sample volume of 2 µL was pipetted onto the optical measuring surface of the lower measurement pedestal. For each sample, three readings were performed to determine the absorbance ratios and concentration. The average value was then calculated to obtain a single measurement for each sample. The same approach was applied to the extraction replicates.

To quantify the level of DNA degradation, samples were further analyzed using the Agilent TapeStation system (Agilent Technologies, Santa Clara, USA). The outputs of the analysis included the DNA integrity number (DIN) and DNA concentration (ng/µL). This analysis was outsourced to STAB VIDA (Almada, Portugal).

### Sequencing application suitability criteria

To assess the suitability of different tissue matrix, preservation method, storage time, to meet quality control criteria for various sequencing techniques, DNA quality control parameters were compiled from different biotechnology service providers. These included concentration (ng/µL), absorbance ratios (260/280 and 260/230), and DNA Integrity Number (DIN). The dataset covered 12 commonly used sequencing techniques: Sanger sequencing, Whole Genome Sequencing (WGS) for both FFPE and non-FFPE samples, PCR-free WGS, Nextera DNA XT, PacBio CLR, PacBio HiFi, Nanopore PromethION, Target Capture (Genomic and FFPE), and Methylation Sequencing (WGBS and RRBS).

As a reference, quality control thresholds were compiled based on publicly available information from sequencing service providers and personal experience from the authors, using such commercial sequencing services. The quality control criteria and corresponding sources are provided in Supplementary Table 4.

Based on the established sequencing quality control criteria, the selection of tissue matrix and preservation method presented in Table 1 was determined through combined analysis of concentration results from both the NanoDrop 1000 spectrophotometer and the Agilent TapeStation system for the criterion “Concentration”, and through combined analysis of the two absorbance ratios (260/280 and 260/230) for the criterion “Purity”. The final selection of tissue matrix, preservation method, or their combination was based on cases where variables showed significant differences in both measurements (NanoDrop and TapeStation for concentration; 260/280 and 260/230 for purity), or where one measurement (i.e. one of the measurement systems, NanoDrop or TapeStation) showed significant differences while the other did not. When no differences were detected using any of the measurement systems, tissue matrices and preservation methods were considered to be equal.

### Statistical analysis

Mixed-effects ANCOVAs were performed to assess the influence of the tissue matrix, DCC, preservation method and storage time on the concentration (ng/µL), on the absorbance measured at 280 nm (260/280) and at 230 nm (260/230), and on the DIN. DNA concentration, 260/280 ratio, and 260/230 ratio were obtained from NanoDrop, while concentration and DIN were measured using TapeStation. In all analyses, NanoDrop values represent the mean of the three measurements performed, while TapeStation values are based on a single measurement provided by the outsourcing service. Tissue matrix, DCC, and preservation method were treated as fixed effects, and storage time was treated as a covariate. The variables were previously tested for normality using the Shapiro-Wilk test and for homogeneity of variances using Levene’s test. All dependent variables were log_10_-transformed before analysis, while storage time was square-root-transformed.

A repeated measures ANOVA, followed by a Dunnett test for specific pairwise differences, was performed to assess significant differences between the initial DNA extraction (control) and DNA extraction replicates in the concentration (ng/µL) and absorbances measured at 280 nm (260/280) and at 230 nm (260/230). The variables were previously tested for normality using the Shapiro-Wilk test and for homogeneity of variances using Levene’s test. Both 260/280 and 260/230 were log_10_-transformed before analysis, whereas DNA concentration was square-root-transformed.

Pearson product-moment correlation coefficients were computed to assess potential linear relationships between the response variables, namely the concentration values (ng/µL), the DIN, and the absorbance measured at 280 nm (260/280) and 230 nm (260/230).

General Discriminant Analysis was performed to evaluate the influence of tissue matrix, preservation method, and storage time on the likelihood of meeting quality control criteria for different sequencing techniques. In this analysis, the tissue matrix and preservation method were treated as categorical independent variables, while storage time was treated as a continuous independent variable. For each sample, success or failure in meeting the criteria was recorded (i.e. according to the criteria in Supplementary Table 4, cases were considered a “success” when the sample DNA value met or exceeded the threshold, and a “failure” when it fell below it), and the percentage of successful outcomes was used as the dependent variable.

The statistical analyses excluded values that were negative for both absorbance and concentration measurements. Negative concentration levels are physically impossible, suggesting potential issues with sampling or DNA extraction processes, and were thus not considered in the analyses.

## Supplementary Information

 Below is the link to the electronic supplementary material.

Supplementary Material 1

Supplementary Material 2

Supplementary Material 3

Supplementary Material 4

Supplementary Material 5

Supplementary Material 6

Supplementary Material 7

## Data Availability

All data supporting the findings of this study are provided in Supplementary Table 1, which contains the raw dataset necessary to reproduce the analyses.
