# Supplementary material for: Evaluating effects of tissue type, preservation method, and decomposition on DNA quality to support genetic sampling in stranded small cetaceans
Source: Sci Rep. 2026 Apr 28;16:13555. doi: 10.1038/s41598-026-41686-x (PMC13121719; doi:10.1038/s41598-026-41686-x)
Supplement: Supplementary file 1 — Supplementary Material 1 [file 41598_2026_41686_MOESM1_ESM.docx]

| ***Sequencing Technique*** | ***Concentration***  ***(ng/ul)*** | ***260/280*** | ***260/230*** | ***DIN*** |
| --- | --- | --- | --- | --- |
| Sanger Sequencing | 2.5 – 40 ^1^ | 1.8 – 2.0 ^1^ | 1.8 – 2.2 ^1^ | ≥ 7 ^2^ |
| WGS (non-FFPE) | 10 ^3^ | 1.8 – 2.0 ^3^ | 1.8 – 2.2 ^1,3^ | ≥ 7 ^4^ |
| WGS (FFPE) | 10 ^3^ | ND | ND | ≥ 4 ^4^ |
| PCR-free WGS | 20 – 30 ^3,5^ | 1.8 – 2.0 ^6^ | 2.0 – 2.2 ^6^ | ≥ 5 ^7^ |
| Nextera DNA XT | 1 ^5,8^ | 1.8 – 2.0 ^8^ | 2.0 – 2.2 ^8^ | ≥ 7 ^5^ |
| PacBio CLR | 50 ^3^ | 1.75 – 2.0 ^3^ | 1.5 – 2.6 ^3^ | ≥ 8 ^4,7^ |
| PacBio HiFi | 50 ^3,7^ | 1.8 – 2.0 ^3^ | 1.5 – 2.6 ^3^ | ≥ 8 ^4,7^ |
| Nanopore Promethion | 100 ^3^ | 1.75 – 2.0 ^3^ | 1.4 – 2.6 ^3^ | ≥ 7 ^7^ |
| Target Capture (Genomic) | 1-20 ^3^ | 1.8 – 2.0 ^3^ | 2.0 – 2.2 ^3^ | ≥ 7 ^5^ |
| Target Capture (FFPE) | 2 ^9^ | ND | ND | ≥ 4 ^4^ |
| Methylation (WGBS) | 10 ^3^ | 1.8 – 2.0 ^10^ | 0 – 3 ^3^ | ND |
| Methylation (RRBS) | 20 ^3^ | 1.8 – 2.0 ^10^ | 0 – 3 ^3^ | ND |

**Table 1.** Sequencing Quality Control Criteria for 12 sequencing techniques. ND – Not determined. Superscript numbers indicate source of information as listed below
