# Supplementary material for: Evaluating effects of tissue type, preservation method, and decomposition on DNA quality to support genetic sampling in stranded small cetaceans
Source: Sci Rep. 2026 Apr 28;16:13555. doi: 10.1038/s41598-026-41686-x (PMC13121719; doi:10.1038/s41598-026-41686-x)
Supplement: Supplementary file 5 — Supplementary Material 5 [file 41598_2026_41686_MOESM5_ESM.docx]

**Supplementary File 1. Raw statistical values of Mixed-effects ANCOVAs with decomposition code, preservation method and matrix treated as fixed effects, and storage days as a covariate**

**ANCOVA Results – Influence of tissue matrix, decomposition condition category (DCC), preservation method and storage time on DNA quality measures**

**Mixed-effects ANCOVAs with decomposition code, preservation method and matrix treated as fixed effects, and storage days as a covariate.**

All dependent variables were log10-transformed, and 'storage days,' was square root-transformed.

**Table 1. Results of the mixed-effects ANCOVA testing the effects of decomposition code, preservation method, and matrix (fixed effects), with storage days as a covariate, on Log₁₀ DIN TS (TapeStation).** df Effect – Degrees of freedom for the tested effect; MS Effect – Mean square of the effect; df Error – Degrees of freedom of the error term; MS Error – Mean square of the error term; F – F-statistic.

|  | Effect | df Effect | MS Effect | df Error | MS Error | F | p-value |
| --- | --- | --- | --- | --- | --- | --- | --- |
| Sqrt Storage days | Fixed | 1 | 0.112542 | 97 | 0.021700 | 5.18619 | **0.024963** |
| 1. Decomposition code | Fixed | 4 | 0.983322 | 97 | 0.021700 | 45.31363 | **0.000000** |
| 1. Preservation method | Fixed | 1 | 0.103014 | 97 | 0.021700 | 4.74713 | **0.031769** |
| 1. Matrix | Fixed | 2 | 0.196941 | 97 | 0.021700 | 9.07549 | **0.000244** |
| (1)*(2) | Fixed | 4 | 0.005956 | 97 | 0.021700 | 0.27400 | 0.894113 |
| (1)*(3) | Fixed | 8 | 0.035081 | 97 | 0.021700 | 1.61662 | 0.129900 |
| (2)*(3) | Fixed | 2 | 0.046315 | 97 | 0.021700 | 2.13430 | 0.123851 |
| (1)*(2)*(3) | Fixed | 8 | 0.019507 | 97 | 0.021700 | 0.89891 | 0.520682 |

**Table 2. Results of the mixed-effects ANCOVA testing the effects of decomposition code, preservation method, and matrix (fixed effects), with storage days as a covariate, on Log₁₀ ng/µL TS (TapeStation).** df Effect – Degrees of freedom for the tested effect; MS Effect – Mean square of the effect; df Error – Degrees of freedom of the error term; MS Error – Mean square of the error term; F – F-statistic.

|  | Effect | df Effect | MS Effect | df Error | MS Error | F | p-value |
| --- | --- | --- | --- | --- | --- | --- | --- |
| Sqrt Storage days | Fixed | 1 | 0.73955 | 114 | 0.231893 | 3.18918 | 0.076786 |
| 1. Decomposition code | Fixed | 4 | 2.74903 | 114 | 0.231893 | 11.85478 | **0.000000** |
| 1. Preservation method | Fixed | 1 | 0.17002 | 114 | 0.231893 | 0.73318 | 0.393650 |
| 1. Matrix | Fixed | 2 | 14.74879 | 114 | 0.231893 | 63.60185 | **0.000000** |
| (1)*(2) | Fixed | 4 | 0.32106 | 114 | 0.231893 | 1.28454 | 0.243725 |
| (1)*(3) | Fixed | 8 | 0.47728 | 114 | 0.231893 | 2.05821 | **0.045682** |
| (2)*(3) | Fixed | 2 | 0.28031 | 114 | 0.231893 | 1.20878 | 0.302358 |
| (1)*(2)*(3) | Fixed | 8 | 0.09245 | 114 | 0.231893 | 0.39867 | 0.919274 |

**Table 3. Results of the mixed-effects ANCOVA testing the effects of decomposition code, preservation method, and matrix (fixed effects), with storage days as a covariate, on Log₁₀ 260/280 ND (NanoDrop).** df Effect – Degrees of freedom for the tested effect; MS Effect – Mean square of the effect; df Error – Degrees of freedom of the error term; MS Error – Mean square of the error term; F – F-statistic.

|  | Effect | df Effect | MS Effect | df Error | MS Error | F | p-value |
| --- | --- | --- | --- | --- | --- | --- | --- |
| Sqrt Storage days | Fixed | 1 | 0.006969 | 113 | 0.001712 | 4.070092 | **0.046019** |
| 1. Decomposition code | Fixed | 4 | 0.001231 | 113 | 0.001712 | 0.718720 | 0.580857 |
| 1. Preservation method | Fixed | 1 | 0.001324 | 113 | 0.001712 | 0.773056 | 0.381139 |
| 1. Matrix | Fixed | 2 | 0.014016 | 113 | 0.001712 | 8.185690 | **0.000479** |
| (1)*(2) | Fixed | 4 | 0.001322 | 113 | 0.001712 | 0.772145 | 0.545534 |
| (1)*(3) | Fixed | 8 | 0.000726 | 113 | 0.001712 | 0.424283 | 0.904386 |
| (2)*(3) | Fixed | 2 | 0.000618 | 113 | 0.001712 | 0.360655 | 0.698019 |
| (1)*(2)*(3) | Fixed | 8 | 0.002338 | 113 | 0.001712 | 1.365710 | 0.219162 |

**Table 4. Results of the mixed-effects ANCOVA testing the effects of decomposition code, preservation method, and matrix (fixed effects), with storage days as a covariate, on Log₁₀ 260/230 ND (NanoDrop).** df Effect – Degrees of freedom for the tested effect; MS Effect – Mean square of the effect; df Error – Degrees of freedom of the error term; MS Error – Mean square of the error term; F – F-statistic.

|  | Effect | df Effect | MS Effect | df Error | MS Error | F | p-value |
| --- | --- | --- | --- | --- | --- | --- | --- |
| Sqrt Storage days | Fixed | 1 | 0.052669 | 115 | 0.014140 | 3.72475 | 0.056075 |
| 1. Decomposition code | Fixed | 4 | 0.025148 | 115 | 0.014140 | 1.77848 | 0.137908 |
| 1. Preservation method | Fixed | 1 | 0.170182 | 115 | 0.014140 | 12.03537 | **0.000736** |
| 1. Matrix | Fixed | 2 | 0.455058 | 115 | 0.014140 | 32.18194 | **0.000000** |
| (1)*(2) | Fixed | 4 | 0.008539 | 115 | 0.014140 | 0.60392 | 0.660585 |
| (1)*(3) | Fixed | 8 | 0.027965 | 115 | 0.014140 | 1.97771 | 0.055304 |
| (2)*(3) | Fixed | 2 | 0.063497 | 115 | 0.014140 | 4.49053 | **0.013249** |
| (1)*(2)*(3) | Fixed | 8 | 0.005255 | 115 | 0.014140 | 0.37160 | 0.933671 |

**Table 5. Results of the mixed-effects ANCOVA testing the effects of decomposition code, preservation method, and matrix (fixed effects), with storage days as a covariate, on Log₁₀ ng/µL ND (NanoDrop).** df Effect – Degrees of freedom for the tested effect; MS Effect – Mean square of the effect; df Error – Degrees of freedom of the error term; MS Error – Mean square of the error term; F – F-statistic.

|  | Effect | df Effect | MS Effect | df Error | MS Error | F | p-value |
| --- | --- | --- | --- | --- | --- | --- | --- |
| Sqrt Storage days | Fixed | 1 | 0.90367 | 114 | 0.137669 | 6.56407 | **0.011710** |
| 1. Decomposition code | Fixed | 4 | 1.20934 | 114 | 0.137669 | 8.78441 | **0.000003** |
| 1. Preservation method | Fixed | 1 | 0.01737 | 114 | 0.137669 | 0.12616 | 0.723103 |
| 1. Matrix | Fixed | 2 | 10.70119 | 114 | 0.137669 | 77.73138 | **0.000000** |
| (1)*(2) | Fixed | 4 | 0.19974 | 114 | 0.137669 | 1.45089 | 0.221887 |
| (1)*(3) | Fixed | 8 | 0.19987 | 114 | 0.137669 | 1.45183 | 0.182803 |
| (2)*(3) | Fixed | 2 | 0.19141 | 114 | 0.137669 | 1.39038 | 0.253171 |
| (1)*(2)*(3) | Fixed | 8 | 0.04350 | 114 | 0.137669 | 0.31594 | 0.958624 |
