# Supplementary material for: Evaluating effects of tissue type, preservation method, and decomposition on DNA quality to support genetic sampling in stranded small cetaceans
Source: Sci Rep. 2026 Apr 28;16:13555. doi: 10.1038/s41598-026-41686-x (PMC13121719; doi:10.1038/s41598-026-41686-x)
Supplement: Supplementary file 6 — Supplementary Material 6 [file 41598_2026_41686_MOESM6_ESM.docx]

**Supplementary File 2. Raw statistical values of a repeated measures Analysis of variance (ANOVA), followed by a Dunnett test (specific pairwise differences) to test whether there were significant differences between first extraction (control) and R1**

**ANOVA Results - DNA extraction variability analysis**

**A repeated measures Analysis of variance (ANOVA), followed by a Dunnett test (specific pairwise differences) was applied to all dependent variables to test whether there were significant differences between first extraction (control) and R1.**

Dependent variables (260/280.ND, 260/230.ND, 260/280.ND.REP, 260/230.ND.REP) were log10 transformed, while ng/µL.ND and ng/µL.ND.REP were square root transformed.

**Table 1. Results of the repeated-measures ANOVA followed by Dunnett’s post hoc test comparing the first extraction (control) with re-extraction (R1) for Log₁₀(260/280 ND – NanoDrop).** SS – Sum of Squares; df – Degrees of freedom; MS – Mean square; F – F-statistic; DCC – Decomposition Condition Category; PM – Preservation Method.

|  | SS | df | MS | F | p-value |
| --- | --- | --- | --- | --- | --- |
| Intercept | 10.79859 | 1 | 10.79859 | 785.0531 | **0.000000** |
| DCC | 0.01250 | 4 | 0.00312 | 0.2272 | 0.922093 |
| PM | 0.09615 | 1 | 0.09615 | 6.9904 | **0.010566** |
| Matrix | 0.03214 | 2 | 0.01607 | 1.1684 | 0.318206 |
| DCC*PM | 0.12842 | 4 | 0.03211 | 2.3341 | 0.066443 |
| DCC*Matrix | 0.11362 | 8 | 0.01420 | 1.0325 | 0.422821 |
| PM*Matrix | 0.07235 | 2 | 0.03618 | 2.6299 | 0.080817 |
| DCC*PM*Matrix | 0.26252 | 8 | 0.03282 | 2.3857 | **0.027176** |
| Error | 0.78405 | 57 | 0.01376 |  |  |
| R1 | 0.00419 | 1 | 0.00419 | 0.4861 | 0.488498 |
| R1*DCC | 0.03094 | 4 | 0.00773 | 0.8964 | 0.472220 |
| R1*PM | 0.07759 | 1 | 0.07759 | 8.9912 | **0.004016** |
| R1*Matrix | 0.00229 | 2 | 0.00115 | 0.1328 | 0.875907 |
| R1*DCC*PM | 0.19799 | 4 | 0.04950 | 5.7360 | **0.000597** |
| R1*DCC*Matrix | 0.28876 | 8 | 0.03610 | 4.1829 | **0.000548** |
| R1*PM*Matrix | 0.11118 | 2 | 0.05559 | 6.4420 | **0.003004** |
| R1*DCC*PM*Matrix | 0.32970 | 8 | 0.04121 | 4.7759 | **0.000159** |
| Error | 0.49187 | 57 | 0.00863 |  |  |

**Table 2. Results of the repeated-measures ANOVA followed by Dunnett’s post hoc test comparing the first extraction (control) with re-extraction (R1) for Log₁₀(260/230 ND – NanoDrop).** SS – Sum of Squares; df – Degrees of freedom; MS – Mean square; F – F-statistic; DCC – Decomposition Condition Category; PM – Preservation Method.

|  | SS | df | MS | F | p-value |
| --- | --- | --- | --- | --- | --- |
| Intercept | 5.215372 | 1 | 5.215372 | 4571.757 | **0.000000** |
| DCC | 0.351419 | 4 | 0.087855 | 77.013 | **0.000000** |
| PM | 0.446971 | 1 | 0.446971 | 391.812 | **0.000000** |
| Matrix | 1.639383 | 2 | 0.819691 | 718.536 | **0.000000** |
| DCC*PM | 0.409379 | 4 | 0.102345 | 89.715 | **0.000000** |
| DCC*Matrix | 0.803320 | 8 | 0.100415 | 88.023 | **0.000000** |
| PM*Matrix | 0.741176 | 2 | 0.370588 | 324.855 | **0.000000** |
| DCC*PM*Matrix | 0.616429 | 8 | 0.077054 | 67.545 | **0.000000** |
| Error | 0.061602 | 54 | 0.001141 |  |  |
| R1 | 0.056372 | 1 | 0.056372 | 39.336 | **0.000000** |
| R1*DCC | 0.026631 | 4 | 0.006658 | 4.646 | **0.002694** |
| R1*PM | 0.013263 | 1 | 0.013263 | 9.255 | **0.003621** |
| R1*Matrix | 0.797516 | 2 | 0.398758 | 278.253 | **0.000000** |
| R1*DCC*PM | 0.150632 | 4 | 0.037658 | 26.278 | **0.000000** |
| R1*DCC*Matrix | 0.074671 | 8 | 0.009334 | 6.513 | **0.000006** |
| R1*PM*Matrix | 0.126730 | 2 | 0.063365 | 44.216 | **0.000000** |
| R1*DCC*PM*Matrix | 0.125342 | 8 | 0.015668 | 10.933 | **0.000000** |
| Error | 0.077386 | 54 | 0.001433 |  |  |

**Table 3. Results of the repeated-measures ANOVA followed by Dunnett’s post hoc test comparing the first extraction (control) with re-extraction (R1) for Sqrt (ng/µL** **ND – NanoDrop).** SS – Sum of Squares; df – Degrees of freedom; MS – Mean square; F – F-statistic; DCC – Decomposition Condition Category; PM – Preservation Method.

|  | SS | df | MS | F | p-value |
| --- | --- | --- | --- | --- | --- |
| Intercept | 11261.22 | 1 | 11261.22 | 311845.6 | **0.000000** |
| DCC | 462.01 | 4 | 115.50 | 3198.5 | **0.000000** |
| PM | 0.11 | 1 | 0.11 | 3.2 | 0.080857 |
| Matrix | 2629.96 | 2 | 1314.98 | 36414.4 | **0.000000** |
| DCC*PM | 266.01 | 4 | 66.50 | 1841.6 | **0.000000** |
| DCC*Matrix | 280.06 | 8 | 35.01 | 969.4 | **0.000000** |
| PM*Matrix | 235.84 | 2 | 117.92 | 3265.5 | **0.000000** |
| DCC*PM*Matrix | 394.07 | 8 | 49.26 | 1364.1 | **0.000000** |
| Error | 2.17 | 60 | 0.04 |  |  |
| R1 | 71.02 | 1 | 71.02 | 1698.0 | **0.000000** |
| R1*DCC | 67.33 | 4 | 16.83 | 402.4 | **0.000000** |
| R1*PM | 12.08 | 1 | 12.08 | 288.8 | **0.000000** |
| R1*Matrix | 214.17 | 2 | 107.09 | 2560.1 | **0.000000** |
| R1*DCC*PM | 93.77 | 4 | 23.44 | 560.4 | **0.000000** |
| R1*DCC*Matrix | 98.70 | 8 | 12.34 | 294.9 | **0.000000** |
| R1*PM*Matrix | 64.32 | 2 | 32.16 | 768.8 | **0.000000** |
| R1*DCC*PM*Matrix | 36.45 | 8 | 4.56 | 108.9 | **0.000000** |
| Error | 2.51 | 60 | 0.04 |  |  |
