# Supplementary material for: Evaluating effects of tissue type, preservation method, and decomposition on DNA quality to support genetic sampling in stranded small cetaceans
Source: Sci Rep. 2026 Apr 28;16:13555. doi: 10.1038/s41598-026-41686-x (PMC13121719; doi:10.1038/s41598-026-41686-x)
Supplement: Supplementary file 7 — Supplementary Material 7 [file 41598_2026_41686_MOESM7_ESM.pdf]

Supplementary Table 1. Raw data file (Excel format) listing all samples and corresponding variables used for statistical and comparative analyses in this study.

Supplementary Table 2. Raw data file (Excel format) listing all samples and corresponding variables from the original DNA extractions and their repetitions, used for statistical and comparative analyses in this study.

Supplementary Table 3. Raw data file (Excel format) listing all General Discriminant Analysis and Pass Rates by Decomposition Condition Categories.

Supplementary Table 4. Sequencing Quality Control Criteria for 12 sequencing techniques. ND – Not determined. Superscript numbers indicate source of information as listed below.

Supplementary File 1. Raw statistical values of Mixed-effects ANCOVAs with decomposition code, preservation method and matrix treated as fixed effects, and storage days as a covariate.

Supplementary File 2. Raw statistical values of a repeated measures Analysis of variance (ANOVA), followed by a Dunnett test (specific pairwise differences) to test whether there were significant differences between first extraction (control) and R1.
